# A Retrospective Analysis of Prevalence of Gastrointestinal Parasites among School Children in the Palajunoj Valley of Guatemala

**DOI:** 10.3329/jhpn.v27i1.3321

**Published:** 2009-02

**Authors:** David M. Cook, R. Chad Swanson, Dennis L. Eggett, Gary M. Booth

**Affiliations:** ^1^ Department of Nutrition, Dietetics, and Food Science, Brigham Young University, 421 N 100 E Provo, UT 84606, USA; ^2^ Johns Hopkins School of Public Health, Utah Valley Regional Medical Center, 1685 N 1590 W Provo, UT 84604, USA; ^3^ Center for Collaborative Research and Statistical Consultation, Brigham Young University, 223 TMCB Provo, UT 84602, USA; ^4^ Department of Plant and Wildlife Sciences, Brigham Young University, 697 WIDB Provo, UT 84602, USA

**Keywords:** Amoebiasis, Ascariasis, *Ascaris lumbricoides*, *Blastocystis hominis*, Child, *Entamoeba histolytica, Giardia lamblia*, Giardiasis, *Hymenolepis nana*, Intestinal diseases, Parasitic, Parasites, Retrospective studies, Guatemala

## Abstract

This study retrospectively analyzed demographic factors that may affect the prevalence of intestinal parasites among Guatemalan school children. The findings of the study showed that young age, wet season, female gender, and severe malnutrition all correlated positively with increased rates of infection. Clinical visits were performed on 10,586 school children aged 5-15 years over a four-year period (2004-2007) in the Palajunoj Valley of Guatemala, during which 5,705 viable stool samples were screened for infection with the following parasites: *Ascaris lumbricoides, Giardia lamblia, Entamoeba histolytica, Hymenolepis nana*, and *Blastocystis hominis.* The average overall prevalences of infection for specific parasites were *A. lumbricoides* 17.7%, *E. histolytica* 16.1%, *G. lamblia* 10.9%, *H. nana* 5.4%, and *B. hominis* 2.8%. Statistical analysis showed significantly higher rates of infection among younger children with *G. lamblia* (odds ratio [OR]=0.905, 95% confidence interval [CI] 0.871-0.941, p<0.0001) and *E. histolytica* (p=0.0006), greater prevalence of *H. nana* among females (OR=1.275, CI 1.010-1.609, p=0.0412), higher infection rates during the wet season for *E. histolytica* (p=0.0003) and *H. nana* (OR=0.734, CI 0.557-0.966, p=0.0275), and greater rates of infection with *G. lamblia* among malnourished children (for moderately malnourished children OR=1.498, CI 1.143-1.963, p<0.0001) and *E. histolytica* (for mildly malnourished children OR=1.243, CI 1.062-1.455, p=0.0313). The results suggest that the prevalence of gastrointestinal parasites among young Guatemalan children is highly dependent on the specific species of the parasite.

## INTRODUCTION

Gastrointestinal parasites contribute significantly to global levels of morbidity and mortality. The World Health Organization (WHO) estimates that over two billion people in the world are infected with at least one form of enteropathogen, the majority of whom reside in developing countries and in areas of poor hygiene (1). School-age children are particularly susceptible to parasitosis, often carrying higher burdens of parasites than adults. According to Brooker *et al.*, the greatest obstacle to effective control of parasites in at-risk populations is inadequate knowledge of the geographical distribution of infection and the demographic variables that influence the prevalence of infection (2). While studies to determine the prevalence of gastrointestinal parasites have been conducted in Guatemala city (3) and in other Latin American countries (4-11), to the best of our knowledge, no study has been undertaken in the Palajunoj Valley or in the Guatemalan Western Highlands.

Nearly half of all Guatemalan children are malnourished, the highest rate among Latin American countries and the fourth highest in the world (12). Poverty in Guatemala is also extreme, with 56% of the population living below the poverty-line and 16% living in extreme poverty (13). Poverty is the greatest among 43% of Guatemalans who are indigenous and who live in rural areas, where 81% of the country's poor and 93% of its extreme poor live. In the Palajunoj Valley, 92.7% of the population is rural, and 95% is indigenous Quiche Maya. The children there, like those living in similar conditions, are vulnerable to increased parasitic infections, malnutrition, stunted development, anaemia, lack of educational opportunities, and child labour (14,15). Aggressive interventions are needed to alleviate the distressed situation in underserved rural areas of Guatemala, such as increased government funding for hospitals and expanded involvement of non-governmental organizations in the health sector.

The health clinic of Primeros Pasos was founded in 2002 to deal specifically with the public-health issues confronting school children in the rural indigenous communities of the Palajunoj Valley. The valley, situated next to the second largest city of Guatemala—Quetzaltenango—is estimated to have 14,481 inhabitants divided among 10 rural communities: Llano del Pinal, Xecaracoj, Las Majadas, Chuicavioc, Tierra Colorada Baja, Tierra Colorada Alta, Xepaché, Candelaria, Bella Vista, and Chuicaracoj. Each year, the clinic invites all children enrolled in each of the 10 community schools to the clinic for a general health screening. Permission was obtained from parents of children to compile health records which have been kept since 2004. This study retrospectively analyzed those records from 2004 to 2007 as these pertain to levels of prevalence of *Ascaris lumbricoides, Giardia lamblia, Entamoeba histolytica, Hymenolepis nana*, and *Blastocystis hominis*. These specific parasites were chosen for study due to their status as the five most commonly-diagnosed parasites at the Primeros Pasos clinic for which medication is required. Data on other parasitic species of interest, such as *Entamoeba coli, Endolimax nana*, and *Trichomonas hominis,* were not recorded with a sufficient rigueur to validate their reporting here, as those species were deemed to be less important to the daily functioning of the clinic.

*A. lumbricoides, G. lamblia, E. histolytica, H. nana*, and *B. hominis* are a significant public-health problem in rural Guatemala and in the developing world as a whole. *A. lumbricoides* infects an estimated 1.472 billion people worldwide, causing morbidity to 335 million and 60,000 deaths annually (16). The helminth disproportionately impacts the health of children, potentially causing malabsorption of nutrients, loss of appetite, impaired growth, vomiting, anaemia, anorexia, and, in extreme cases, death through intestinal obstructions (17,18). *G. lamblia* is among the most common intestinal protozoa in the world, infecting more than 200 million people worldwide, although it is markedly more prevalent in developing countries, including Guatemala (19). Infection requires ingestion of as few as 10 viable cysts and is easily spread through contamination of piped water. Painful gas, bloating, fatty diarrhoea, and general epigastric pain are frequently associated with infection. *E. histolytica* is the major cause of amoebic dysentery in the world, infecting an estimated 480 million people worldwide and causing about 70,000 deaths annually (20). Symptoms of infection include abdominal pain, bloody stools, severe diarrhoea, weight loss, and fatigue, and fatality may occur if the amoeba reaches the liver and is left untreated. The helminth *H. nana* infects nearly 75 million people worldwide (16). Symptoms of infection include abdominal pain, loss of appetite, itching around the anus, irritability, and diarrhoea. Little is known for certain of the epidemiology of *B. hominis*, although it is becoming more accepted as a causative agent of disease (21). *B. hominis* has a worldwide distribution and is most prevalent in areas of low socioeconomic status. Symptoms associated with infection range from abdominal pain and constipation to diarrhoea, vomiting, fatigue, nausea, and fever.

The aim of this study was to identify demographic variables potentially associated with increased rates of gastrointestinal parasite infection among the school children of the Palajunoj Valley of Guatemala. It is hoped that the results of this study will help improve the effectiveness of the Primeros Pasos clinic in designing and promoting its health initiatives in the valley and in aiding other public-health policy-makers active in the Guatemalan Highlands. The data will also contribute to the overall understanding of the epidemiology of *A. lumbricoides, G. lamblia, E. histolytica, H. nana*, and *B. hominis.*

## MATERIALS AND METHODS

### Subjects

According to 2004 census data gathered by the Guatemalan Ministry of Public Health and Social Assistance, there were 5,228 children, aged 5-15 years, living in the 10 communities of the Palajunoj Valley (2,666 males, 2,562 females) (22). Of the 5,228 children, 2,968 (56.8%) were enrolled in public schools; 2,697 (90.9%) of the children enrolled in public schools were seen by the Primeros Pasos clinic; 1,108 (41.1%) of them provided usable stool samples (Table 1). The clinic saw 10,586 children aged 5-15 years during 2004-2007; 5,705 (53.9%) children brought viable stool samples. The study excluded subjects who were not aged 5-15 years and/or who did not provide a viable stool sample.

**Table 1.** Summary of the number of children seen by the clinic and the percentage of children who brought viable stool samples in 10 communities of the Palajunoj Valley

Community	No. of children from each school seen by clinic					% of children who brought viable stool samples			
	2004	2005	2006	2007		2004	2005	2006	2007
Bella Vista	81	81	85	84		12.3	28.4	∗	42.9
Candelaria	147	126	149	154		∗	42.1	49.7	41.3
Chuicaracoj	31	32	31	35		93.5	96.9	93.6	2.8
Chuicavioc	289	243	249	296		66.1	54.3	50.2	77.4
Las Majadas	200	236	233	238		33.5	32.6	38.0	61.3
Llano del Pinal	811	864	862	751		74.4	73.0	74.5	70.3
Tierra Colorada Alta	122	99	99	137		∗	64.6	56.6	40.8
Tierra Colorada Baja	241	175	179	179		∗	82.9	70.9	79.3
Xecaracoj	596	602	553	525		23.5	52.8	37.4	65.0
Xepachè	179	198	201	193		69.8	65.7	56.7	66.3
Total	2,697	2,656	2,641	2,592	Overall %	41.1	54.4	55.2	65.3

∗Data incomplete or missing

The Institutional Review Board for human subjects at Brigham Young University, Provo, Utah, approved the study.

### Processing of samples

Health workers from the clinic visited the classrooms of students scheduled for clinic-visits one to two days before their appointments to explain the importance of bringing a stool sample and to distribute small plastic containers with instructions on how to gather faeces in the morning of the day of their visit. A volunteer collected stool samples of children at the clinic; stool samples were labelled and taken to the in-house laboratory for analysis. These were processed immediately upon collection using a standard saline wet mount procedure. Samples for which identification of parasites was difficult or uncertain were stained with an iodine solution (1% I_2_ and 2% KI) to facilitate analysis. A trained pathologist screened the slides for eggs of *A. lumbricoides* or *H. nana*, cysts of *E. histolytica, B. hominis*, or *G. lamblia*, trophozoites of *G. lamblia*, or amoebas of *E. histolytica,* and the results were recorded on an Excel spreadsheet.

### Data processing

The height, weight, age, and sex of each child was measured and recorded. The severity of malnutrition was calculated using the child's weight-for-age ratio, following the guidelines of the World Health Organization (23). Due to the high incidence of stunting among Guatemalan children, weight-for-age was deemed to be a more appropriate measure of malnutrition than standard height-for-weight ratios. Children with weight-for-age ratios at 90% or above the mean of the reference population were assigned a level of ‘normal’ malnutrition while those between 75% and 89.9% were assigned a level of ‘mild’ malnutrition, 60% to 74.9% were categorized as moderately malnourished, and those at 59.9% or below were considered to be severely malnourished.

### Interventions

A qualified physician or a health professional fluent in Spanish physically examined the children. After their check-up, the children attended classes on parasite prevention, hand-washing, dental care, and basic nutrition. Appropriate medications were administered once a year for any parasites found. Children diagnosed with infection due to *A. lumbricoides* were given a single dose of 400 mg albendazole while those diagnosed with *H. nana* were given a single dose of niclosamide dependent on body-weight (1.5 g for children weighing more than 34 kg, 1 g for children weighing between 11 and 34 kg). Children diagnosed with *E. histolytica, G. lamblia*, and *B. hominis* were given metronidazole in weight-dependant dosages (for *E. histolytica*: 35-50 mg metronidazole per kg of body-weight in three doses a day for 7-10 days; for *G. lamblia*: 15 mg metronidazole per kg of body-weight in two doses a day for 5-7 days; and for *B. hominis*: 15-30 mg metronidazole per kg of body-weight in three doses a day for 10 days). Medications were administered as per the protocols recorded in the 2003 edition of the Sanford Guide to Antimicrobial Therapy (24).

### Statistical analysis

The gathered data were carefully vetted, and incomplete data points were discarded. Logistic regression analyses (using the SAS software, version 9.1) of the presence or absence of each parasite type was performed. The independent variables that were used in the models were age, year, season, gender, and malnutrition status as main effects, along with all two-way interactions with these variables. For each parasite, the model was reduced by eliminating all interactions that had a p value of >0.15 through backward elimination, leaving a model with all main effects and significant two-way interactions. A p value of <0.05 was considered significant.

## RESULTS

Viable stool samples were collected from 5,705 (53.9%) of the 10,586 children seen by the clinic from 2004 to 2007. Table 1 presents the percentages of children from each school providing samples each year. Years and schools that had incomplete or missing data were excluded from the study.

The overall prevalence of parasitic infection by year was recorded, with the results presented in the figure. A. *lumbricoides* was the most commonly-found parasite in 2004, with 33.1% of the children testing positive for infection. The prevalence of *A. lumbricoides* declined over the years, and by 2007, *E. histolytica* and *G. lamblia* had become the most prevalent parasites with 18.1% and 13.3% of the children infected respectively; 15.1% (n=167) were infected with two or more parasites in 2004, a rate which dropped to 6.7% (n=97) in 2005, 6.5% (n=96) in 2006, and 5.3% (n=89) in 2007. In the largest school—Llano del Pinal—the drop among children with multiple parasite infections was even more pronounced: 14.9% (n=91) in 2004, 7.5% (n=42) in 2005, 4.3% (n=24) in 2006, and 1.9% (n=10) in 2007.

**Fig.**
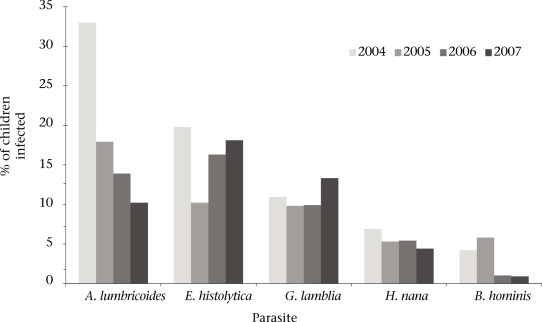
Summary of parasitic infection rates among school children in the Palajunoj Valley for 2004–2007

### Specific parasites

A logistic regression analysis was performed on individual parasitic infection to examine associations between infection and the child's age, gender, and malnutrition status and the season of the year (wet or dry) in which infection occurred. Results for specific parasites are summarized in Table 2 and 3, which present pertinent odds ratios and model parameter estimates respectively.

**Table 2.** Summary of odds ratios and confidence intervals

Characteristics	*G. lamblia*	*B. hominis*	*H. nana*	*A. lumbricoides*	*E. histolytica*
	OR (95% CI)	OR (95% CI)	OR (95% CI)	OR (95% CI)	OR (95% CI)
Gender (comparison level=male)					
Female	0.925 (0.782-1.093)	∗	1.275 (1.010-1.609)	∗	∗
Age (comparison level=age (years))					
+ 1 year	0.905 (0.871-0.941)	∗	0.964 (0.915-1.015)	∗	∗
Season (comparison level=wet)					
Dry	∗	∗	0.734 (0.557-0.966)	∗	∗
Survey year (comparison level=2007)					
2006	∗	∗	1.284 (0.928-1.777)	∗	∗
2005	∗	∗	1.330 (0.950-1.863)	∗	∗
2004	∗	∗	1.479 (1.053-2.079)	∗	∗
Malnutrition level (comparison level=normal)					
Mild	1.149 (0.956-1.381)	0.957 (0.674-1.359)	0.994 (0.773-1.279)	∗	1.243 (1.062-1.455)
Moderate	1.498 (1.143-1.963)	1.314 (0.800-2.158)	1.191 (0.822-1.726)	∗	1.152 (0.907-1.464)
Severe	4.201 (1.701-10.377)	2.196 (0.297-17.305)	<0.001 (<0.001->999.999)	∗	0.461 (0.108-1.979)

∗No odds ratios to report for variables involved in significant two-way interactions; CI=Confidence interval; OR=Odds ratio

**Table 3.** Summary of model parameter estimates for individual logistic regressions

Characteristics	*G. lamblia*	*B. hominis*	*H. nana*	*A. lumbricoides*	*E. histolytica*
Intercept	−0.9178	−2.9725	−5.2774	−1.2722	−2.5729
Gender (comparison level=male)					
Female	−0.0390	0.0575	0.1213	−0.0270	−0.0086
Age (comparison level=age (years))					
+ 1 year	−0.0996	−0.0691	−0.0396	−0.0133	0.0595
Season (comparison level=wet)					
Dry	−0.1591	0.0254	−0.1547	−0.0259	−0.6996
Survey year (comparison level=2007)					
2006	−0.0085	0.6003	0.0183	−0.3691	0.1503
2005	−0.0401	0.3154	0.0535	−0.1909	−0.5340
2004	−0.1271	1.3904	0.1600	1.5447	0.1486
Malnutrition level (comparison level=normal)					
Mild	−0.3556	−0.2981	2.6942	−0.0666	0.3209
Moderate	−0.0906	0.0193	2.8746	0.0200	0.2454
Severe	0.9408	0.5327	−8.2688	0.2492	−0.6699
Age ∗ season (comparison level=age (years) ∗ wet)					
+ 1 year ∗ Dry	†	†	†	†	0.0659
Age ∗ year (comparison level=age (years) ∗ 2007)					
+ 1 year ∗ 2006	†	−0.1582	†	†	†
+ 1 year ∗ 2005	†	0.0783	†	†	†
+ 1 year ∗ 2004	†	−0.0709	†	†	†
Season ∗ gender (comparison level=wet ∗ male)					
Dry ∗ Female	†	−0.2227	†	†	†
Year ∗ season (comparison level=2007 ∗ wet)					
2006 ∗ Dry	0.0932	†	†	−0.3093	−0.0467
2005 ∗ Dry	0.1743	†	†	0.3414	0.2604
2004 ∗ Dry	−0.1165	†	†	0.7275	−0.1660
Year ∗ gender (comparison level=2007 ∗ male)					
2006 ∗ Female	†	†	†	0.0761	−0.00187
2005 ∗ Female	†	†	†	0.0680	−0.1677
2004 ∗ Female	†	†	†	−0.1406	0.1011

^†^No parameters reported for non-significant two-way interactions

Higher rates of infection due to *G. lamblia* were observed among younger children (p<0.0001) and among children with more severe levels of malnutrition (p=0.0009). *G. lamblia* was one of two parasites that exhibited significantly higher rates of infection among younger children and malnourished children, the other being *E. histolytica* in both cases*.* Children with a severe level of malnutrition were, on average, 4.2 times more likely to be infected with *G. lamblia* cysts. *H. nana*-associated infection was found to be greater among females (p=0.0412) and during the wet season (p=0.0275). *H. nana* was the only parasite with significantly higher rates of infection in either gender and one of two parasites that showed greater rates of infection during the wet season, the other being *E. histolytica.* The p value of 0.0412 associated with a higher rate of infection in females for *H. nana* is only nominally significant, and the authors are unaware of any variation in lifestyle habits or gender-specific environmental conditions which would explain the observed higher rates of *H. nana*-associated infection in females. Infections due to *E. histolytica* were greater during the wet season (p=0.0003), among younger children (p=0.0006), and among children with more severe levels of malnutrition (p=0.0313). *E. histolytica* was one of two parasites associated with each of these significant findings, the others being *H. nana* for the wet season and *G. lamblia* for both younger age and greater levels of malnutrition.

In addition to the demographic variables tested, a significant decrease in infection rate over the four-year course of the programme was also observed in three of the five parasites. *A. lumbricoides* (p<0.0001), *E. histolytica* (p<0.0001), and *B. hominis* (p=0.0298) all had significantly reduced rates of prevalence in 2007 versus 2004.

### Demographics

The Guatemalan Ministry of Public Health and Social Assistance in 2004 gathered demographic data for each community (22). Workers went from house to house with a questionnaire to gather information on population size and distribution, number of homes per community, disposition of available potable water, and common methods of sewage disposal. For our purposes, ‘adequate’ sewage disposal was defined as a flush toilet, a latrine, or other defined areas for placing excrement that limited its contact with the surrounding environment and potential for contamination of soil and food with faeces. Table 4 presents a summary of data collected.

**Table 4.** Summary of 2004 demographic information for 10 communities of the Palajunoj Valley

Community	Total no. of population	Average children per household	% of homes with potable water	% of homes with adequate sewage disposal
Llano Del Pinal	5,181	11.31	84.7	82.8
Xecaracoj	4,556	10.62	68.5	78.1
Las Majadas	1,593	11.46	47.5	64.7
Chuicavioc	1,395	11.43	80.3	77.9
Tierra Colorada Baja	917	7.91	46.6	69.8
Tierra Colorada Alta	489	8.15	0.0	31.7
Xepaché	298	11.04	74.4	84.5
Candelaria	287	3.02	43.2	70.5
Bella Vista	226	5.38	90.5	71.4
Chuicaracoj	161	6.19	30.8	73.1

## DISCUSSION

### Specific parasites

Our findings highlight the epidemiological variability and complexity of each parasite examined. The prevalence of *A. lumbricoides* in Latin America and throughout the world has been reported at various levels in recent years: 41.4% in Chongqing province of China in 2003 (25), 40.5% in Cuba in 2007 (9), 25.7% in Malaysia in 2007 (26), 15% in Sierra Leone in 2007 (27), and 3.8% in rural Argentina in 2002-2003 (10). The prevalence of *A. lumbricoides* among school children of the Palajunoj Valley dropped from 33.1% to 10.2% over the four-year course of the programme. A similar decrease in infection due to *A. lumbricoides—* from 63.2% to 17% in one year—as a result of clinical intervention was described in a study done in Turkey (28). Moist soils and relative atmospheric humidity are associated with healthier *A. lumbricoides* populations while a dry climate is hostile to their development (2). Our study, however, did not find higher rates of infection during the wet season for *A. lumbricoides* (p=0.9809).

The prevalence of *G. lamblia* was reported at 25% in Cuba in 2007 (9), 17.6% in Malaysia in 2007 (26), and 6.9% in rural Argentina in 2002-2003 (4). The prevalence of *G. lamblia* among school children in the Palajunoj Valley, averaged 10.9% between 2004 and 2007. The results of our research indicate that both younger children and children with severe malnutrition had greater levels of infection (p=<0.0001 and p=0.0009 respectively).

Risk factors for infection with *E. histolytica* are unclear at present and mostly speculative in nature. A study in rural Mexico reported *E. histolytica*-associated infection rates of over 50% (29) while, in the Palajunoj Valley, the prevalence averaged 16.1% between 2004 and 2007. In our study, infection due to *E. histolytica* was more common among younger children (p=0.0006) during the wet season (p=0.0003) and among severely-malnourished children (p=0.0313). The prevalence of infection due to *E. histolytica* also decreased significantly over the course of the programme (p<0.0001) as did the incidence of parasitic infection in other studies (30).

Little is known for certain of the epidemiology of *B. hominis*. Developing countries generally have a higher rate of infection (30-50%) than developed countries (1.5-10%). The prevalence of *B. hominis* was 33.5% in Cuba in 2007 (9), 8.1% in Malaysia in 2007 (26), and 27.2% in rural Argentina in 2002-2003 (4). The prevalence of *B. hominis* in the Palajunoj Valley decreased from 4.2% in 2004 to 0.9% in 2007—unusually low levels for a developing country. This may be due, in part, to our exclusive consideration of children as significantly higher rates of infection have been reported in adults compared to children (7,31). A study in rural Argentina found rates of infection due to *B. hominis* to be correlated with inadequate latrine-use (4), and studies in Nepal and Egypt found that *B. hominis*-associated infection was more common during the hot and dry periods of the year (32,33). Our study, however, found no significant variation in infection rates by season of the year (p=0.9046)—an observation also reported elsewhere (34,35). Infections were found to peak between 10 and 14 years of age in Spain in 1992 (36) while our study did not find significant associations of age with *B. hominis*-associated infection (p=0.9759). No significant difference in infection due to *B. hominis* was observed between genders in our study (p=0.8345), which agrees with data reported elsewhere (6,21,37).

The prevalence of *H. nana*-associated infection in the Palajunoj Valley decreased from 6.9% in 2004 to 4.4% in 2007. Infections were greater during the wet seasons (p=0.0275) and higher among females (p=0.0412). A study in Cuba, however, did not find seasonal variance in infection due to *H. nana* and found that males had higher rates of infection than females (11).

### Demographics

The differing demographics of the 10 communities of the Palajunoj Valley may have some bearing on the rates of infections observed in each community. Pronounced variation exists among the communities in their degrees of access to potable water and adequate sewage disposal and in the average number of children per household. This variation can be related to rates of infection due to *A. lumbricoides, E. histolytica,* and *G. lamblia*—the three most common parasites in the valley. Chuicavioc had the highest prevalence of *A. lumbricoides* among any community, with a 47.6% average infection rate over the four years of the study versus a mean of 17.8% across all schools. The incidence of infection with multiple parasites was also the highest in Chuicavioc (16.7% versus a mean of 7.0% for all schools). This may be related to the high density of children among families in the community (11.46 children per household). Children from Las Majadas had higher rates of infections due to *E. histolytica* and *G. lamblia* when compared with all schools (23.7% and 22.2% respectively compared to means of 13.2% and 10.5%). This may be related to a high percentage of homes without readily-available potable water (52.5%) which likely resort to obtaining water from sources that are contaminated.

### Limitations

The methods employed for gathering stool samples subject the study to some bias in the selection of children. Primeros Pasos only recruited children who were attending public schools in the valley, and as of 2004, only 56.8% of children aged 5-15 years were enrolled in school. Data were not gathered on truancy levels for the remaining three years of the study nor on the overall population of 5-15 years old in the valley. The number of children enrolled in public schools, however, remained relatively steady from 2004 to 2007 (Table 1). In 2004, younger children were more likely to attend school, and truancy levels increased with greater age: in 2003, 85% of children aged 7-12 years were enrolled in the first grade, but only 40% of children reached the fourth grade, and less than 33% finished the sixth grade. Many children assist their families in the fields or at home instead of attending classes, a practice common in rural Guatemala. The percentage of children attending school who participated in the health programme was quite high (90.9% in 2004). However, not all children who participated in clinical visits brought viable stool samples, and no sample-randomization techniques were employed. As Table 1 shows, 41.1% of children attending the clinic brought stool samples, with 54.4%, 55.2%, and 65.3% bringing samples in 2005, 2006, and 2007 respectively.

A large number of the same children were seen during the four-year span of the study, with children turning 16 years being excluded and those turning five years included. Patients who received treatment in previous years were not excluded from the study. There is a high likelihood that the children were initially treated successfully for any infections found. Similar multiple doses, 5-10 days of treatments with metronidazole for children with giardiasis have been found to cure infections at a median rate of 94% across eight studies (38). Treatment with albendazole for ascariasis in children of KwaZulu-Natal, South Africa, was 96.4% effective, with re-infection rates of approximately 40% over a 29-week period (39). Parasitic infections in the Palajunoj Valley should be studied in future to determine the incidence of re-infections.

The simple smear and iodine-staining techniques employed for collecting and processing stool samples do not yield data on parasite loads but only on the prevalence of parasites. The methods were chosen for their efficiency and cost-effectiveness but are less sensitive than other commonly-used procedures. Additional techniques, such as formalin-acetate concentration or simple sedimentation, would need to be used for gathering useful data on the relative intensity of infections.

In conclusion, the results of the study suggest that the prevalence of parasitic infections among young Guatemalan children is highly dependent on the parasite species. Young age, wet season, female gender, and severe malnutrition all correlated positively with increased rates of parasitic infection for at least one of the five parasites examined but no single variable was associated with more than two parasites. Malnutrition was associated with increased rates of infection for *G. lamblia* and *E. histolytica* only while infection during the wet season was higher for *H. nana* and *E. histolytica* only. Age was significant only in infections due to *G. lamblia* and *E. histolytica*, and infection in females was only greater for *H. nana*. Many of our findings supported those of previous studies, although some did not. Future research may be conducted using more comprehensive diagnostic techniques to determine not only the prevalence of infection but also the intensity and relative parasite loads of the study population.

## ACKNOWLEDGEMENTS

The authors gratefully acknowledge Jessica Ohana Gonzalez, Director of the Primeros Pasos Clinic, for her support and advice throughout this project and Brigham Young University's Office of Research and Creative Activities for their financial support.
